# Does Pre-Operative Education Reduce Length of Stay and Improve Clinical Outcomes After Elective Orthopaedic Surgery? A Systematic Review and Meta-Analysis

**DOI:** 10.7759/cureus.99369

**Published:** 2025-12-16

**Authors:** Oliver J Negus, Joshua B V. Smith

**Affiliations:** 1 Trauma and Orthopaedics, Norfolk and Norwich University Hospitals NHS Foundation Trust, Norwich, GBR; 2 Trauma and Orthopaedics, James Paget University Hospitals NHS Foundation Trust, Great Yarmouth, GBR

**Keywords:** arthroplasty, education, elective orthopaedics, health education, joint replacement, joint school, orthopaedics, physical education, prehabilitation, pre-operative education

## Abstract

The aim of this review was to assess whether pre-operative patient education can reduce hospital length of stay and improve clinical outcomes for adult patients undergoing elective orthopaedic operations. The study was registered on the NIHR PROSPERO database (ID: CRD42021224356) and conducted according to the PRISMA guidelines. A search of electronic databases in February 2023 found 17 studies. The quality of evidence was assessed using the GRADE approach. A meta-analysis was conducted, and the outcomes were presented as mean difference, standardised mean difference, or a risk ratio. Pre-operative patient education offered benefit with a shorter length of stay by a mean of 0.37 days (n=1,729; 95% CI: -0.65 to -0.09; p=0.01) and these results were corroborated by sensitivity analyses studying RCTs only (MD: 0.42; 95% CI 0.77 to 0.07; p=0.02) and studies deemed to be at low risk of bias (MD: 0.24; 95% CI 0.44 to 0.04; p=0.02). Pre-operative patient education also offered a benefit to patient satisfaction (SMD: 0.43; 95% CI: 0.01 to 0.84; p=0.04) and improved short-term psychological scores (SMD: -0.44; 95% CI: -0.82 to -0.06; p=0.02). It offered no benefit for pain scores, functional scores, frequency of adverse events, and quality of life, and these results failed to reach statistical significance. Pre-operative patient education is associated with a decreased length of hospital stay and improved patient satisfaction and short-term psychological scores.

## Introduction and background

The development of patient education as an important part of clinical practice and research originated in the 1960s and 1970s. Prior to this, doctors were considered to have the sole responsibility for the diagnosis and management of patients’ conditions [1]. Early education focused on giving patients information, often via booklets and videotapes [2]. In the 1990s, researchers started to focus on using education to empower patients to make shared decisions about healthcare [3,4]. In the present day, widespread access to the internet has allowed patients to educate themselves on their conditions; the so-called Dr Google approach to healthcare education [5].

Patient education is an established part of orthopaedic practice, including enhanced recovery programmes [6,7] and joint schools [8,9]. There is evidence that patient education may improve pre-operative knowledge of the procedure and recovery [8]. This may lead to earlier inpatient discharge due to lower anxiety levels and improved patient satisfaction associated with better expectations management. Pre-operative assessment and patient education are currently recommended in the National Institute for Health and Care Excellence (NICE) guidelines for joint replacement and the Enhanced Recovery After Surgery Society recommendations for perioperative care in THR and total knee replacement (TKR) surgery [10,11]. Other than the 2014 Cochrane review, which suggested a shorter length of stay following total knee replacement [12], current systematic reviews have failed to find significant evidence that pre-operative education impacts post-operative outcomes [12-14].

A study in the United Kingdom suggested that over one-third of patients on waiting lists for THR and nearly one-quarter waiting for TKR have a quality of life worse than death [15]. In addition, waiting times for elective orthopaedic surgery have been exacerbated by the COVID-19 pandemic [16]. Any intervention that can reduce the length of stay may reduce the chances of patients being cancelled on the day of surgery due to a lack of beds, thus allowing surgical teams to treat more patients.

The aim of this study was to assess whether pre-operative patient education can reduce hospital length of stay and improve clinical outcomes, such as patient satisfaction, psychological scores, pain scores, functional scores, adverse events, and quality of life, for adult patients undergoing elective orthopaedic surgical procedures.

This article was previously presented as a poster presentation and a virtual oral presentation at the 2022 British Orthopaedic Trainees Association (BOTA) Congress on November 25, 2022.

## Review

A systematic review was conducted in accordance with the Preferred Reporting Items for Systematic Reviews and Meta-Analysis (PRISMA) statement checklist. The review was prospectively registered on PROSPERO with registration number CRD42021224356 [17].

A PICOS (Population, Intervention, Comparison, and Outcome) strategy was used to set the search strategy and eligibility criteria [18]. The population was adults aged 18 and over undergoing elective orthopaedic surgical procedures. For the purposes of this review, we included spinal and hand surgery as orthopaedic procedures, even if carried out by allied specialties. Intervention was defined as any educational intervention in which a predominant feature is an enhanced level of patient education regarding the anaesthetic, surgery, and recovery period. Comparison was defined as a maximum of basic pre-operative education only, given by the surgeon or a pre-assessment healthcare professional. The primary outcome was length of stay, with secondary outcomes of patient satisfaction, pain scores, functional scores, adverse events, quality of life, and psychological scores, at a maximum of 18 months post-operatively. Study designs included were randomised and non-randomised experimental trials and epidemiological studies. Synonyms associated with the PICOS strategy that were used to develop the search strategy are shown in Table 1.

**Table 1:** Table showing elements of PICOS used to create the search strategy.

Population	Intervention	Comparison	Outcomes	Study design
Orthopaedics, Orthopedics, Arthroplasty, Replacement, Spinal surgery, Joint surgery, Spinal operation, Joint operation, Decompression, Orthopedic procedures, Traumatology	Education, Teaching, Taught, Learning, Information, Joint school, Pre-habilitation, Prehabilitation, Class	Not applicable (search terms related to the comparison were not used in the search strategy)	Not applicable (search terms related to the outcomes were not used in the search strategy)	Trial, Randomised, Randomized, Observational

OVID MEDLINE, OVID EMBASE, CINAHL, AMED, and PsycINFO were the online electronic databases used for the search. WHO Clinical Trials Registry and ClinicalTrials.gov were also searched. The search was performed from 1st January 1990 (or inception if more recent) to 28th February 2023. Search terms were adapted depending on the database being used. The search term “replacement” was removed from the search strategy after a pilot search of OVID MEDLINE returned multiple studies looking at electrolyte replacement. Search strategies for each database are available in Appendix A.

Due to time and resource constraints, only English-language studies were included. Studies were deemed eligible if they met the following criteria: patients aged 18 years and over undergoing elective orthopaedic surgical procedures, for any reason; the study compares pre-operative patient education with no formal pre-operative patient education; the study is an experimental or epidemiological/observational comparative study; the study measures length of stay, patient surgical satisfaction, pain scores, functional scores, adverse events, quality of life, and/or psychological outcomes. Any studies with a retraction statement were excluded from the review; however, the study citation was documented for potential discussion. The eligibility criteria checklist is available in Appendix B. The authors independently selected the studies to be included by reviewing titles, abstracts, and full texts, with discrepancies solved by consensus decision. Citations were stored in EndNote (Clarivate Analytics, USA).

Data extracted from the studies meeting the eligibility criteria included: author, year of publication, study design, number of participants (in each arm), population characteristics, details of intervention, details of comparison, and outcomes (including all reported statistical data). Outcome data were categorised as short-term (0 to 5 days), medium-term (6 to 28 days), or long-term (29 days or longer). Corresponding authors were contacted by the lead author when data were missing from the written report.

Methodological quality and risk of bias were assessed by the authors independently, with discrepancies solved by consensus decision. Risk of bias in randomised-controlled trials (RCTs) was assessed using the Cochrane Risk of Bias 2 (RoB 2) tool [19]. Risk of bias in non-randomised trials was assessed using the Risk of Bias in Non-randomised Studies - of Interventions (ROBINS-I) tool [20].

Meta-analysis was performed where possible for the primary and secondary outcomes using RevMan v5.4 (Cochrane Collaboration, UK). Mean difference (MD) was used for the length of stay (time in days). Standardised mean difference (SMD) was used for other continuous data, which was reported with different scoring systems. Adverse events data were dichotomous, so the risk ratio was used. For the continuous outcomes, the data were meta-analysed using inverse-variance. For the dichotomous outcomes, the Mantel-Haenszel method was used. Throughout, 95% confidence intervals were reported. A random effects model was used, and statistical heterogeneity was assessed by the I² statistic. Forest plots were produced to graphically illustrate the meta-analyses. Sensitivity analyses of RCTs only and studies deemed to have a low risk of bias were also performed to further evaluate the primary outcome, length of stay. Grading of Recommendations Assessment, Development and Evaluation (GRADE) [21] was used to assess the quality of the overall body of evidence. Where there was substantial study heterogeneity or insufficient data to permit meta-analysis, a narrative analysis was performed.

**Figure 1:**
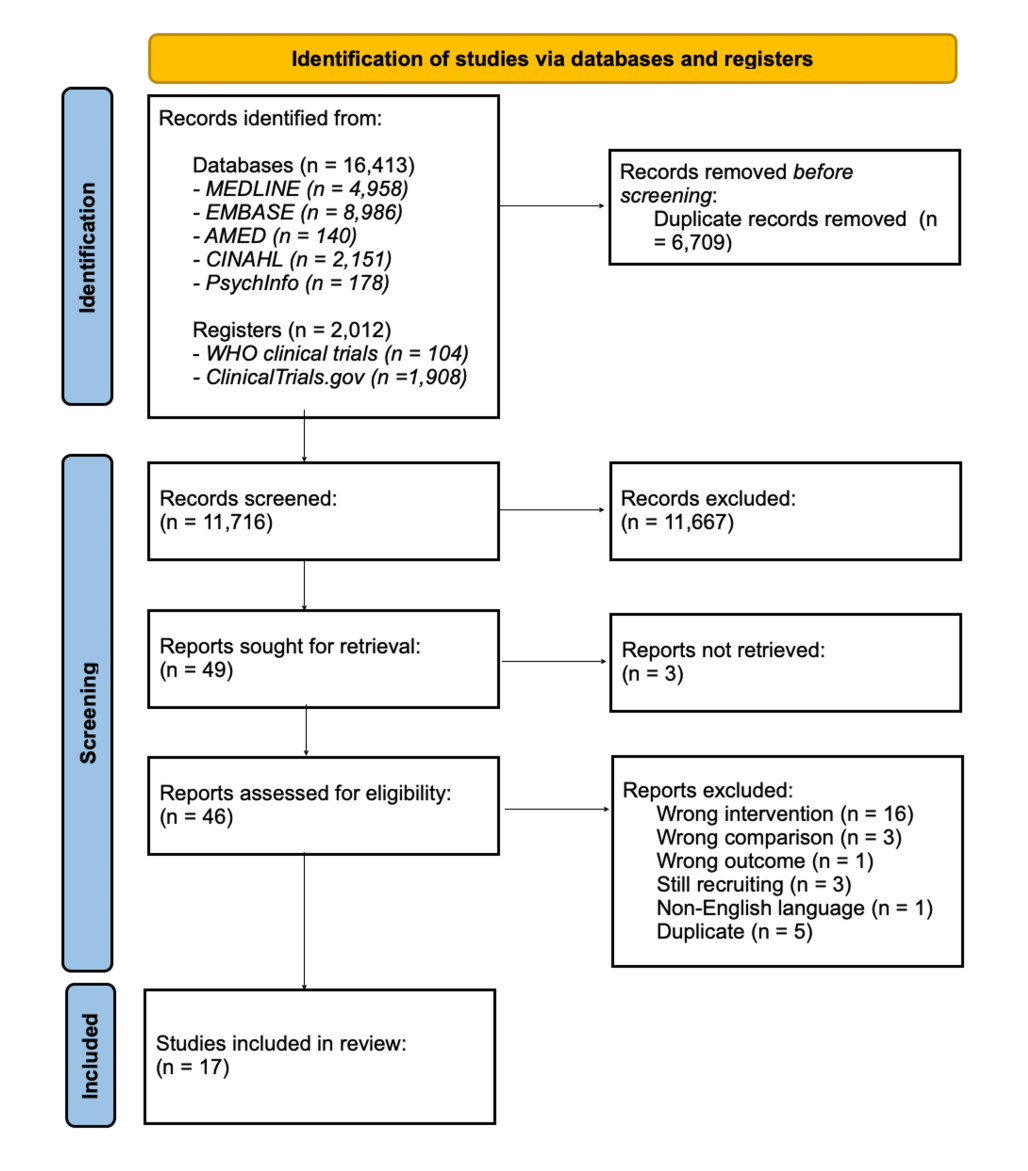
PRISMA flow diagram

16,413 records were identified from database searches, and 2,012 records from clinical trials registries. 17 studies were included within the systematic review and meta-analysis. The PRISMA flowchart is shown in Figure 1.

Table 2 shows the study characteristics and a summary of the findings. The 17 studies involved a total of 2,357 participants; 1,177 in intervention groups and 1,180 in comparison groups. 12 studied patients undergoing lower limb arthroplasty [22-33], four studied patients undergoing spinal surgery [34-37], and one studied patients undergoing arthroscopic shoulder surgery [38]. Thirteen studies were RCTs, and four were non-randomised studies.

**Table 2:** Summary of included studies and their findings. RCT: randomised control trial; THR: total hip replacement; BMI: body mass index; VAS: visual analogue scale; TKR: total knee replacement; WOMAC: Western Ontario and McMaster Universities Osteoarthritis Index; ASA: American Society of Anaesthesiologists score; MDT: multidisciplinary team.

Study ID	Author/Year	Type of study	Number of participants Intervention/comparison (total)	Population characteristics	Intervention	Comparison	Summary of findings
1	Biau et al. 2018 [22]	RCT	103/96 (199)	40-90 years old undergoing primary THR for osteoarthritis; BMI ≤30	Small group (<3 patients) class with physiotherapist and nurse. Taught about pain management and exercises for rehabilitation	Usual care – optional large group (>10) information session	Length of stay 7 days in both groups (p=0.66); 4 complications in each group (p=1); mean VAS pain score at day three improved by 1 point in the intervention group (p=0.26)
2	Clode et al. 2018 [23]	Prospective cohort study	52/23 (75) (Participants self-selected their preferred cohort)	Healthy patients undergoing primary THR or TKR for osteoarthritis	Twice weekly 15-minute education sessions covering perioperative issues, prehabilitation exercises, and usual care	Usual care – information booklet and 1 hour group discussion	No significant difference in pain, WOMAC or quality of life scores; Median length of stay was three days in both cohorts
3	Crowe and Henderson 2003 [24]	RCT	65/68 (133)	Elective THR or TKR (excluding operations for cancer, revision or 2^nd^ joint operation in <2 years)	Information video and booklet; information on length of stay, rehabilitation programme and diet; tour of the surgical ward; demonstration of rehabilitation equipment; individualised counselling from an occupational therapist; and usual care	Usual care – 7-hour pre-assessment clinic visit	Length of stay significantly reduced in intervention group (6.55 days versus 10.50 days; p=0.032); Fewer complications in intervention group (7 versus 22; p=0.007)
4	Giraudet-Le Quintrec et al. 2003 [25]	RCT	48/52 (100)	<80 years old undergoing elective primary THR for osteoarthritis; ASA ≤2	Small group (3-6 patients) MDT teaching; presentation slides; and usual care	Usual care – verbal information from the surgeon and anaesthetist, and an information booklet	No significant differences in pain scores, anxiety, complications, length of stay or patient satisfaction
5	Hoppe et al. 2014 [38]	RCT	20/20 (40)	<18 years old undergoing arthroscopic rotator cuff or labral repair	Video teaching and usual care	Usual care – pre-operative consultation with the surgeon	Satisfaction scores remained similar (9.3 versus 9.5; p=0.445)
6	Soeters et al. 2018 [26]	RCT	63/63 (126)	18-85 years old undergoing unilateral THR or TKR	Physiotherapist-led education session; access to online rehabilitation microsite	Usual care – group education class	No significant difference in length of stay; WOMAC score improved in the intervention group (6 versus 14; p<0.001)
7	Huang et al. 2018 [27]	RCT	126/117 (243)	Unilateral primary TKR for osteoarthritis	Prehabilitation exercises; information on perioperative journey; information booklet; pre-admission phone call from physiotherapist to answer any questions	Usual care – no formal educational programme	Average length of stay improved by one day (p=0.027); no significant differences in pain scores or complications
8	Kesanen et al. 2017 [34]	RCT	50/50 (100)	>18 years old undergoing surgery for spinal stenosis	Formative knowledge test; individualised feedback and information session with a nurse via telephone; and usual care	Usual care – routine consultations with nurse and surgeon	No significant difference in length of stay; mean pain score improved in the intervention group (26.97 versus 33.91; significance not reported)
9	Lee et al. 2018 [35]	RCT	42/40 (82)	Age ≥20 undergoing lumbar spine surgery	20-page information booklet; videos; pictures; and usual care	Usual care – five to ten-minute discussion with nurse	Length of stay not significantly improved (6.71 versus 7.03; p=0.06); anxiety and pain scores significantly improved in intervention group (p=0.009 and p<0.001 respectively)
10	Lee et al 2020 [36]	Prospective cohort study	63/124 (187)	>20 years old undergoing surgery for cervical myelopathy due to ossification of the posterior longitudinal ligament	Information booklets, video, and two separate discussions with the surgeon	Usual care – a single discussion with the surgeon	Significant improvement in patient satisfaction score (7.8 versus 7.1; p=0.024); no significant differences in pain, functional or psychological scores; no significant difference in complications
11	Marques et al. 2021 [28]	Retrospective observational study	335/341 (676)	Patients undergoing primary THR or TKR	One-hour MDT teaching session, with detailed information on perioperative journey	No formal education session	No difference in length of stay (10.3 days versus 10.6 days; p=0.6); Improved functional and quality of life scores (p=0.004 and p=0.02, respectively)
12	Eschalier et al. 2017 [29]	RCT	22/20 (42)	55-75 years old undergoing TKR for osteoarthritis	Standardised information booklet; and usual care	Usual care – oral information from the surgeon	No significant difference in length of stay (9.6 versus 8.8; p-value not documented)
13	Percope de Andrade et al. 2021 [30]	RCT	45/34 (79)	>45 years old undergoing primary TKR for osteoarthritis	MDT lecture series on perioperative journey	Usual care – standard surgical consultation	No significant differences in pain, functional or quality of life scores
14	Vukomanovic et al. 2008 [31]	RCT	23/22 (45)	>70 years old undergoing primary THR for osteoarthritis	Information and a brochure from a physiatrist, and exercises from a physiotherapist	No formal education or pre-operative exercises	No significant difference in length of stay (9.8 days versus 10.2 days; p=0.62); no significant differences in pain or functional scores
15	Wilson et al. 2016 [32]	RCT	73/70 (143)	Patients undergoing primary TKR; ASA ≤2	Information booklet; individualised teaching session; follow-up phone call to answer questions; and usual care	Usual care: Physiotherapist outlining rehabilitation activities; 30-minute video about the planned operation; review of patient-controlled analgesia	No significant difference in length of stay (3.7 days versus 4.5 days; p=0.06); no significant difference in pain or quality of life scores
16	Feninets et al. 2022 [37]	RCT	23/17 (40)	>18 years old undergoing spinal decompression surgery for disc herniation or lumbar stenosis	Oral briefing lasting 30-45 minutes, and an information leaflet from an experienced nurse	Usual care: standard care from healthcare providers	Pain scores lower in the intervention group (p=0.037); no significant difference in psychological scores
17	Pinskiy et al. 2021 [33]	Non-randomised parallel-group controlled trial	24/23 (47)	Patients undergoing their first primary THR	Oral briefing and presentation by physiotherapist; information booklet; and usual care	Usual care: meeting with surgeon, anaesthetist and nurse	Improved psychological, functional and satisfaction scores in the intervention group (all p<0.01); no significant difference in length of stay or pain scores

**Figure 2:**
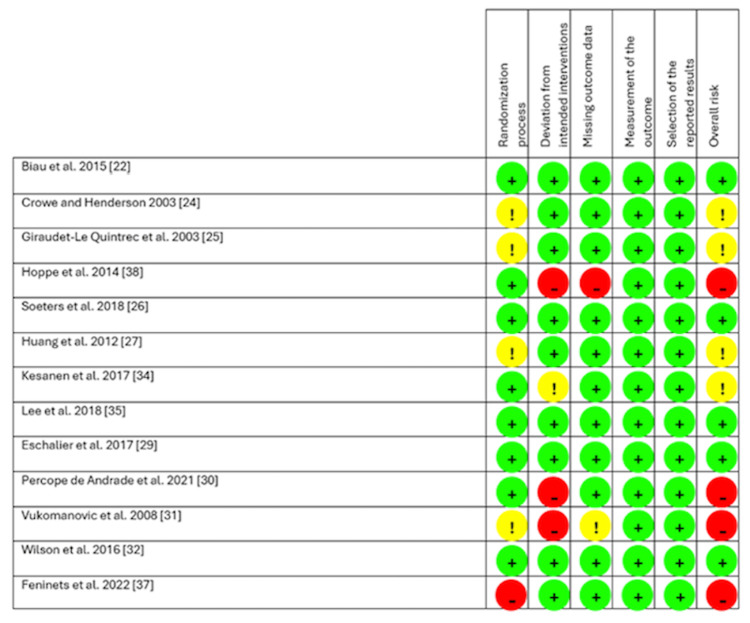
Risk of bias in RCTs using Cochrane RoB 2. Green '+': low risk; yellow '!': some concerns; red '-' : high risk.

**Figure 3:**
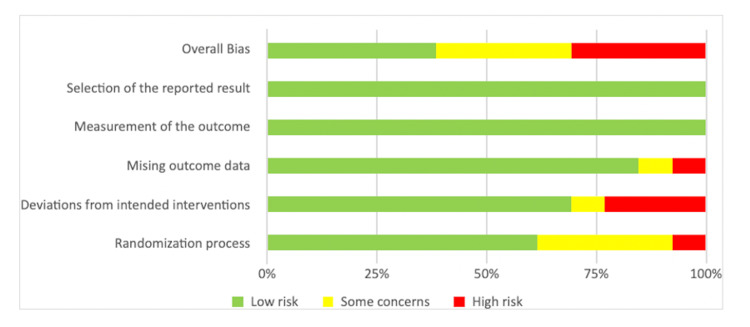
Summary of risk of bias in RCTs using Cochrane RoB 2.

With the exception of two studies [28,31], all studies used usual care as a comparison and gave their own definition of this (Table 2). In all included studies, the level of patient education exceeded the usual care, with the level of intervention ranging from standardised information booklets to multidisciplinary teaching sessions and tours of surgical wards. 13 studies reported data for the primary outcome, length of stay [22-29, 31-35]. The risk of bias was deemed to be low for five of the 13 RCTs as per the RoB-2 tool. Four studies were deemed to have some concerns, and four had a high risk of bias. The RoB-2 findings are summarised in Figures 2, 3.

**Figure 4:**
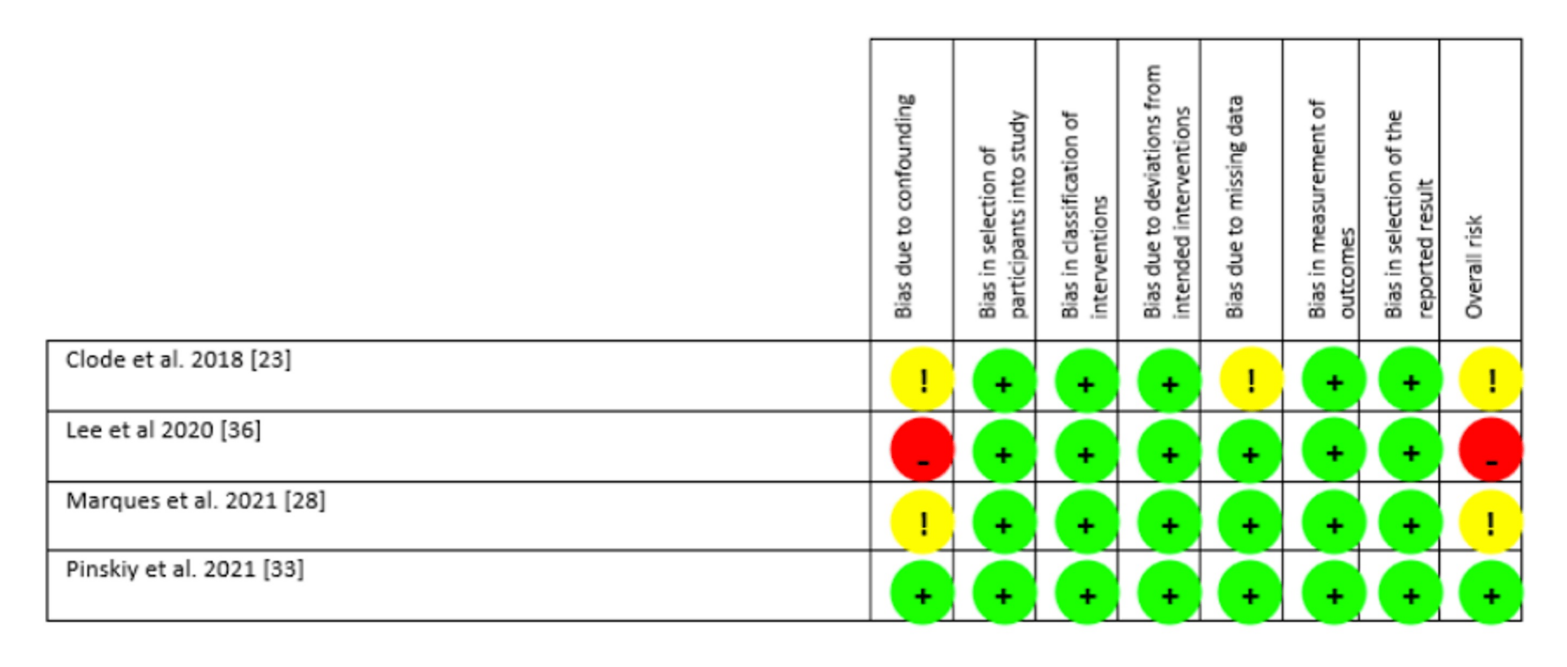
Risk of bias in non-randomised studies using ROBINS-I Green '+': low risk; yellow '!': some concerns; red '-': high risk.

**Figure 5:**
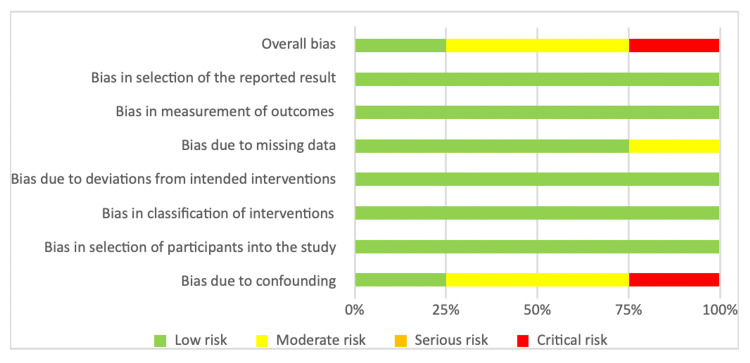
Summary of risk of bias in non-randomised studies using ROBINS-I.

The risk of bias was deemed to be low for one non-randomised study and moderate for two studies as per the ROBINS-I tool. The other non-randomised study was deemed to be at critical risk of bias due to confounding. This particular study compared one surgeon who used pre-operative education with two other surgeons who did not use pre-operative education. The ROBINS-I findings are summarised in Figures 4, 5.

Length of stay

**Figure 6:**
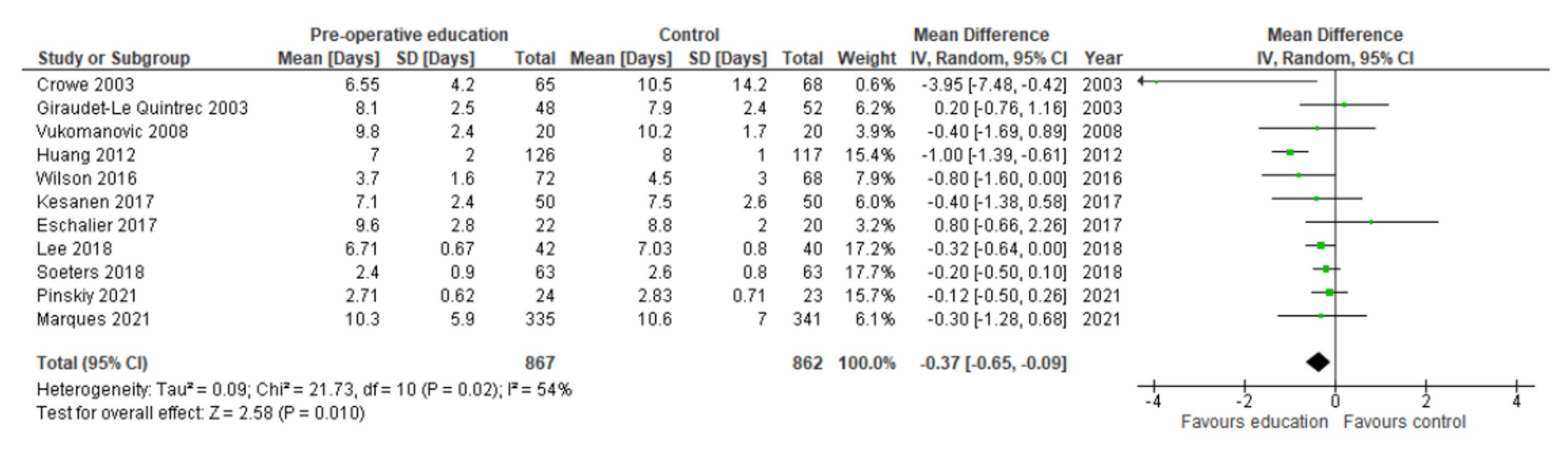
Forest plot showing results of meta-analysis for length of stay. References (top to bottom): Crowe 2003 [24], Giraudet-Le Quintrec 2003 [25], Vukomanovic 2008 [31], Huang 2012 [27], Wilson 2016 [32], Kesanen 2017 [34], Eschalier 2017 [29], Lee 2018 [35], Soeters 2018 [26], Pinskiy 2021 [33], Marques 2021 [28].

11 studies reported sufficient data for quantitative analysis of length of stay [24-29, 31-35]. Two studies did not report the required statistical data for inclusion in quantitative analysis [22,23]. Attempts to contact the corresponding authors were made with no success. All included studies measured length of stay in days. Patient education offered a benefit with a shorter length of stay by a mean of 0.37 days (MD: 0.37; 95% CI 0.65 to 0.09; N=1729; I²=54%; p=0.010). The forest plot is shown in Figure 6.

GRADE assessment found the evidence to be of moderate certainty. It is therefore quite probable that reduced length of stay following pre-operative education is close to the estimated effect size of 0.37 days. The GRADE assessment is summarised in Table 3.

**Table 3:** GRADE assessment of the quality of evidence for the length of stay (primary) outcome quantitative analysis.

Number of studies	Certainty assessment						Number of patients		Effect	Quality of evidence
	Study design	Risk of bias	Imprecision	Inconsistency	Indirectness	Other considerations	Pre-operative education	Control		
Primary outcome: length of stay										
11	9 RCTs 1 non-RCT 1 observational study	Not serious	Not serious	Serious (I^2^=54%)	Not serious	No evidence of publication bias	867	862	Shorter mean length of stay in education group by 0.37 days (95% CI: 0.65 to 0.09)	Moderate ⊕⊕⊕O

One study that could not be included in the meta-analysis included a total of 199 participants; 103 in the educational intervention group and 96 in the control group [22]. This study found an average length of stay of seven days in both groups (p=0.66). The other study stated a median length of stay of three days in both groups [23].

Sensitivity Analyses, Length of Stay

**Figure 7:**
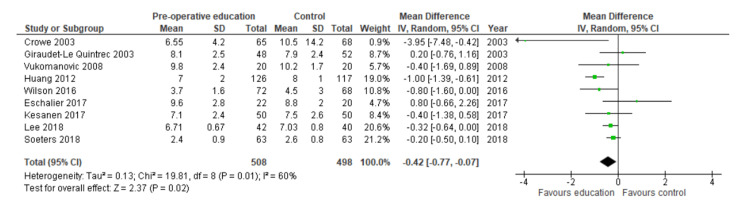
Forest plot showing results of sensitivity analysis for length of stay in RCTs. References (top to bottom): Crowe 2003 [24], Giraudet-Le Quintrec 2003 [25], Vukomanovic 2008 [31], Huang 2012 [27], Wilson 2016 [32], Eschalier 2017 [29], Kesanen 2017 [34], Lee 2018 [35], Soeters 2018 [26].

An RCT-only sensitivity analysis found that patient education offered a benefit with a shorter length of stay by a mean of 0.42 days (MD: 0.42; 95% CI 0.77 to 0.07; N=1006; I=60%; p=0.02). The forest plot is shown in Figure 7.

**Figure 8:**
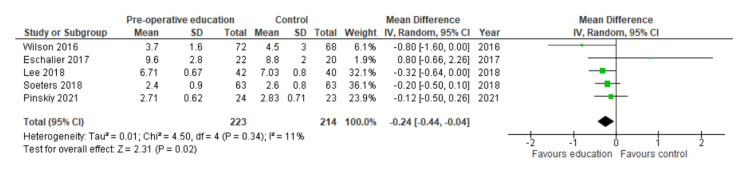
Forest plot showing results of sensitivity analysis for length of stay in studies deemed to have low risk of bias. References (top to bottom): Wilson 2016 [32], Eschalier 2017 [29], Lee 2018 [35], Soeters 2018 [26], Pinskiy 2021 [33].

A sensitivity analysis of the studies deemed to be at low risk of bias found that patient education offered a benefit with a shorter length of stay by a mean of 0.24 days (MD: 0.24; 95% C: I0.44 to 0.04; N=437; I²=11%; p = 0.02). The forest plot is shown in Figure 8.

Patient satisfaction

**Figure 9:**
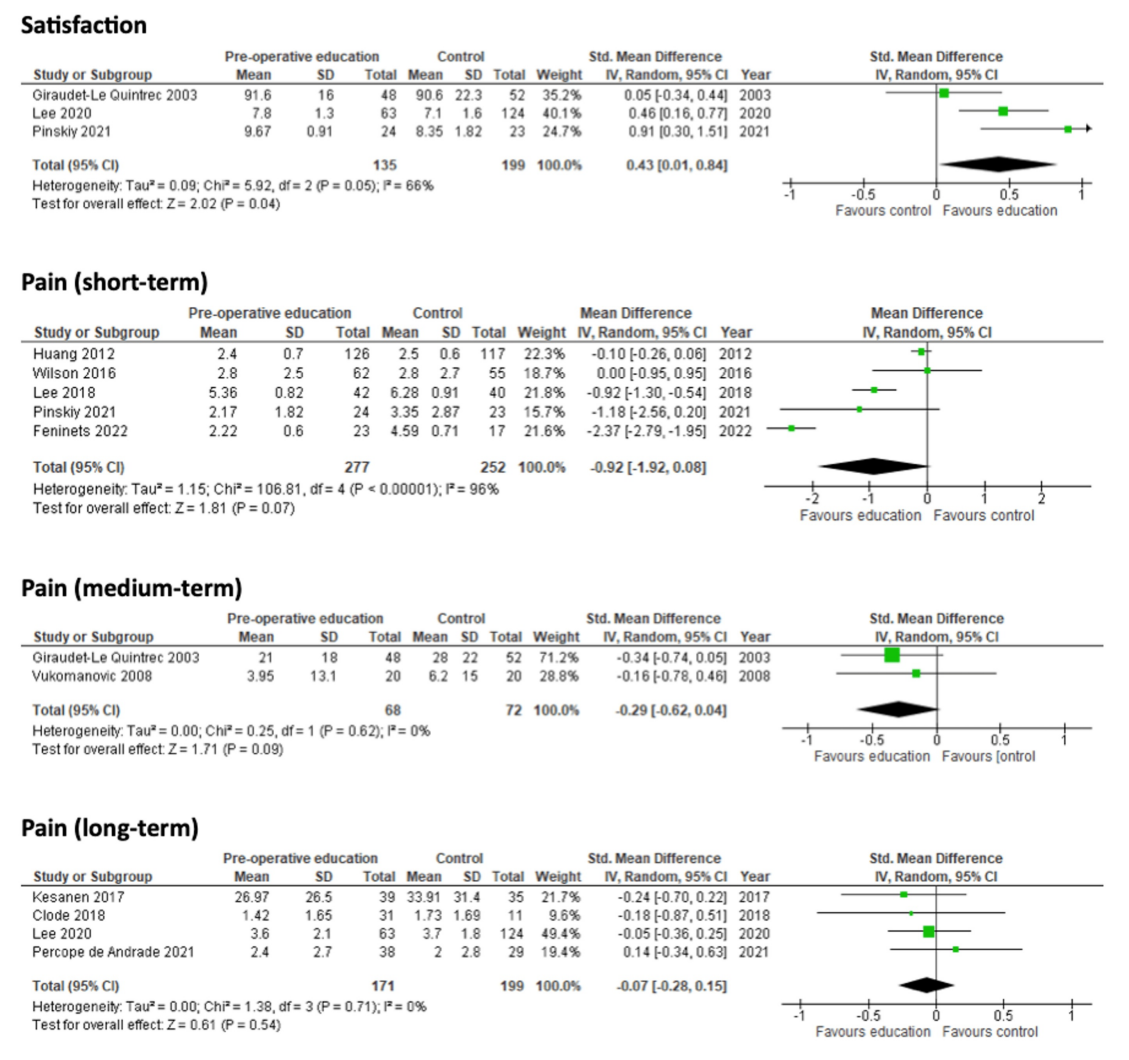
Forest plots for the secondary outcomes of satisfaction, pain (short-term), pain (medium-term) and pain (long-term). References (top to bottom). Satisfaction outcome: Giraudet-Le Quintrec 2003 [25], Lee 2020 [36], Pinskiy 2021 [33]. Pain (short-term) outcome: Huang 2012 [27], Wilson 2016 [32], Lee 2018 [35], Pinskiy 2021 [33], Feninets 2022 [37]. Pain (medium-term) outcome: Giraudet-Le Quintrec 2003 [25], Vukomanovic 2008 [31]. Pain (long-term) outcome: Kesanen 2017 [34], Clode 2018 [23], Lee 2020 [36], Percope de Andrade 2021 [30].

Four studies reported results for post-operative patient satisfaction [25,33,36,38]. One study did not report sufficient statistical data for inclusion in the quantitative analysis [38]. An email was sent to the corresponding author, but no response was received. Of the included studies, two measured patient satisfaction on a 0-10 scale [33,36], with the other study measuring patient satisfaction from 0%-100% [25]. Patient education offered a benefit to improve patient satisfaction compared to usual care (SMD: 0.43; 95% CI: 0.01 to 0.84; N=334; I²=66%; p=0.04) as shown in the forest plot in Figure 9.

GRADE assessment found the evidence to be of low certainty. GRADE assessments for the secondary outcomes can be seen in Table 4.

**Table 4:** GRADE assessments for the secondary outcomes.

Number of studies	Certainty assessment						Number of patients		Effect	Quality of evidence
	Study design	Risk of bias	Imprecision	Inconsistency	Indirectness	Other considerations	Pre-operative education	Control		
Secondary outcome: Patient satisfaction										
3	1 RCT, 1 non-randomised trial, 1 observational study	Serious	Not serious	Serious	Not serious	No evidence of publication bias	135	199	Improved patient satisfaction (SMD: 0.43; 95% CI: 0.01 to 0.84)	Low ⊕⊕OO
Secondary outcome: Pain scores (short-term, medium-term and long-term)										
5	4 RCT, 1 observational study	Not serious	Serious	Very serious	Not serious	No evidence of publication bias	277	252	No benefit to pain scores in the short-term (MD: -0.92; 95% CI: -1.92 to 0.08)	Very low ⊕OOO
2	2 RCT	Serious	Serious	Not serious	Not serious	No evidence of publication bias	68	72	No benefit in the medium-term (SMD: -0.29; 95% CI: -0.62 to 0.04)	Low ⊕⊕OO
4	2 RCT, 2 observational study	Very serious	Serious	Not serious	Not serious	No evidence of publication bias	171	199	No benefit in the long-term (SMD: -0.07; 95% CI: -0.28 to 0.15)	Very low ⊕OOO
Secondary outcome: Functional abilities										
7	3 RCT, 1 non-randomised trial, 3 observational studies	Very serious	Serious	Very serious	Not serious	No evidence of publication bias	574	613	No benefit to functional abilities (SMD: -0.32; 95% CI: -0.71 to 0.07)	Very low ⊕OOO
Secondary outcome: Adverse events										
6	5 RCT, 1 observational study	Serious	Serious	Serious	Not serious	No evidence of publication bias	477	525	No benefit in reducing the frequency of adverse events (RR: 0.79; 95% CI: 0.44 to 1.39)	Very low ⊕OOO
Secondary outcome: Quality of life										
3	1 RCT, 2 observational studies	Serious	Very serious	Serious	Not serious	No evidence of publication bias	404	383	No benefit to long-term quality of life (SMD: 0.01; 95% CI: -0.78 to 0.80)	Very low ⊕OOO
Secondary outcome: Psychological scores (short-term)										
5	4 RCT, 1 non-randomised trial	Not serious	Not serious	Serious	Not serious	No evidence of publication bias	185	178	Benefit to short-term psychological scores (SMD: -0.44; 95% CI: -0.86 to -0.06)	Moderate ⊕⊕⊕O

The study excluded from the meta-analysis included 34 participants; 15 in the intervention group and 19 in the control group [38]. The control group reported slightly higher satisfaction scores, with a mean score of 9.5, compared to 9.3 in the intervention group; however, this result was not statistically significant (p=0.445).

Pain scores

Pain scores were reported in 12 studies [22,23,25,27,30-37]. Of these, six reported pain scores for the short-term (zero to five days) [22,27,32-33,35,37]; two for the medium-term (six to 28 days) [25, 31]; and four for the long-term (29 days, or later) [23,30,34,36]. One study reported insufficient statistical data for short-term pain scores [22], leaving five studies included in the meta-analysis. Patient education offered no benefit to pain scores in the short-term (MD: -0.92; 95% CI: -1.92 to 0.08; N=529; I²=96%; p=0.07). There was no benefit in the medium-term (SMD: -0.29; 95% CI -0.62 to 0.04; N=140; I²=0%; p=0.09). There was also no benefit in the long-term (SMD: -0.07; 95% CI: -0.28 to 0.15; N=370; I²=0%; p=0.54). The forest plots are shown in Figure 9. GRADE assessment found the evidence to be of low to very low certainty.

Functional scores

**Figure 10:**
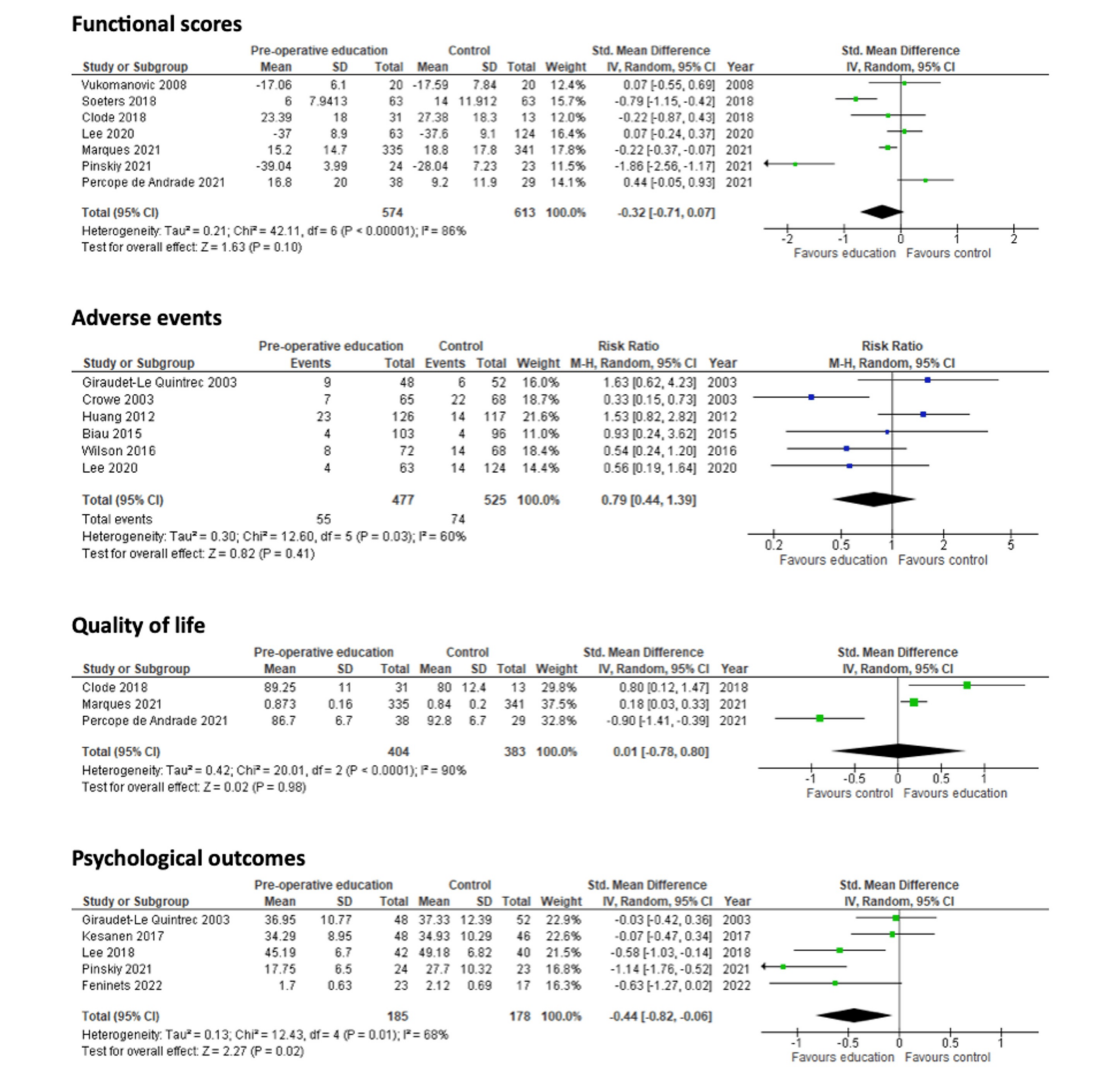
Forest plots for the secondary outcomes of functional scores, adverse events, quality of life and psychological outcomes. References (top to bottom). Functional scores outcome: Vukomanovic 2008 [31], Soeters 2018 [26], Clode 2018 [23], Lee 2020 [36], Marques 2021 [28], Pinskiy 2021 [33], Percope de Andrade 2021 [30]. Adverse events outcome: Giraudet-Le Quintrec 2003 [25], Crowe 2003 [24], Huang 2012 [27], Biau 2015 [22], Wilson 2016 [32], Lee 2020 [36]. Quality of life outcome: Clode 2018 [23], Marques 2021 [28], Percope de Andrade 2021 [30]. Psychological outcome: Giraudet-Le Quintrec 2003 [25], Kesanen 2017 [34], Lee 2018 [35], Pinskiy 2021 [33], Feninets 2022 [37].

Seven studies reported results for functional scores [23,26,28,30,31,33,36]. Four studies measured functional scores using the Western Ontario and McMaster Universities Arthritis Index (WOMAC) [23,26,28,30], two used the Oxford Hip Score (OHS) [31,33], and one used the physical component score of the Short-Form 36 (SF-36) [36]. Patient education offered no benefit to functional scores (SMD: -0.32; 95% CI: -0.71 to 0.07; N=1,187; I²=86%; p=0.10). The forest plot is shown in Figure 10.

GRADE assessment found the evidence to be of very low certainty.

Adverse events

Six studies reported the frequency of adverse events [22,24,25,27,32,36]. Patient education offered no benefit in reducing the frequency of adverse events (RR: 0.79; 95% CI: 0.44 to 1.39; N=1,002; I²=60%; p=0.41). The forest plot is shown in Figure 10. GRADE assessment found the evidence to be of very low certainty.

Quality of life

Three studies reported results for long-term quality of life [23,28,30]. Two studies measured quality of life using the EuroQol EQ5D [23,28], whilst one study used the 36-Item Short Form Survey (SF-36) [30]. Patient education offered no benefit to long-term quality of life (SMD: 0.01; 95% CI: -0.78 to 0.80; N=787; I²=90%; p=0.98). The forest plot is shown in Figure 10. GRADE assessment found the evidence to be of very low certainty.

Psychological scores

Six studies reported results for psychological scores [25,33-37]. Four studies reported short-term results only [33-35,37], one study reported both short and medium-term results [25], and one study reported long-term results [36]. The studies reporting medium-term and long-term psychological scores did not find any statistically significant differences between the intervention and control groups [25,36]. Patient education offered benefit to short-term psychological scores (SMD: -0.44; 95% CI: -0.82 to -0.06; N=363; I²=68%; p=0.02). The forest plot is shown in Figure 10. GRADE assessment found the evidence to be of moderate certainty.

The results of this review suggest that pre-operative patient education is associated with a modest decrease in length of hospital stay following elective orthopaedic surgery in adults, compared with no formal pre-operative education. This finding was corroborated by two sensitivity analyses. The results also suggest that patient education may improve post-operative patient satisfaction and anxiety.

This result builds upon the systematic review by Johansson et al., which identified two studies with reduced length of stay after pre-operative education, but did not have sufficient data to perform a meta-analysis [13]. It also builds upon the 2014 Cochrane review looking at pre-operative education in lower limb arthroplasty [12]. This review found suggestions that it may decrease the length of stay following hip replacement by 0.79 days, but this was not statistically significant. The same review did, however, find a statistically significant shorter length of stay following knee replacement by 1.86 days, in favour of pre-operative education.

The evidence for the effects of pre-operative education on length of stay appears to be mixed in other surgical specialities, with reduced length of stay following bariatric surgery [39], but no significant findings following cardiac [40] or major abdominal surgery [41]. We suspect that this may be related to the more predictable nature of some elective procedures, such as gastric band surgery, compared with less predictable procedures such as gastrointestinal tract cancer resection. Much of elective orthopaedic surgery is predictable, especially primary joint replacement surgery, and this allows for patient education to be tailored to the exact procedure taking place.

In the education group, patient satisfaction was greater (p=0.04) and post-operative anxiety levels were reduced (p=0.02). Statistically significant results were not found for the other secondary outcomes. These findings appear to be in keeping with the rest of the literature. The review by Johansson et al. [13] also suggested reduced anxiety following surgery in the intervention group, but lacked statistical significance. The review by Aydin et al. [42] suggested a reduction in pre-operative anxiety. We believe that by allowing patients to understand what to expect, they become less anxious in the short-term and are therefore more confident to be discharged sooner. There is further evidence to suggest that patients with lower pre-operative anxiety levels have improved mood and reduced pain levels in the post-operative period [43]. Our results, however, may overestimate the effect of patient education on improving patient satisfaction as the GRADE assessment found the evidence to be of low certainty, and the outcome was underpowered (N=334). 

The main limitations in this review article include the moderate-to-high heterogeneity in multiple outcomes and the risk of bias. As highlighted in the GRADE assessment, there was statistical heterogeneity in the analysis of the primary outcome, length of stay (I²=54%), leading to serious inconsistency. This was also seen in the sensitivity analysis for RCTs only (I²=60%). However, the sensitivity analysis of the studies deemed to be at low risk of bias found that patient education offered a benefit with a shorter length of stay by a mean of 0.24 days (p=0.02), and these results had low heterogeneity (I²=11%). Heterogeneity levels were only low in medium-term and long-term pain score secondary outcomes. We conducted rigorous risk of bias assessments for the RCTs and non-randomised studies; however, another limitation is that four RCTs had a high risk of bias, and one non-randomised study had a critical risk of bias. There is also the theoretical risk of publication bias.

The studies included in this review article covered lower limb arthroplasty, shoulder arthroscopy and spinal procedures; however, because not all elective orthopaedic procedures were covered, it may be difficult to generalise the findings of this review to the entirety of elective orthopaedic surgery. The review process itself was limited by its exclusion of non-English language studies. This may have led to important data not being included in the final analysis, especially from studies with non-significant findings that failed to get published in a major English-language journal.

Whilst a reduced length of stay by 0.37 days may not sound like a dramatic finding, it may have positive implications for the future of elective orthopaedic surgery. From the perspective of improving patient outcomes, shorter length of stay results in a decreased risk of healthcare-associated infections and a decreased risk of side effects from medications [44]. Furthermore, it could be theorised that reducing the length of stay may come with additional financial benefits. In the United Kingdom, it costs on average £536 per 24 hours for a patient to occupy a National Health Service hospital ward bed [45]. It is therefore likely that reducing length of stay by the adoption of pre-operative patient education would be both clinically and financially justified.

Future research into the secondary outcomes assessed in this review is needed to fully understand the benefits of pre-operative patient education. Meta-regressions and subgroup analyses will also be useful because of the moderate-to-high heterogeneity seen in this review article. Multi-arm RCTs comparing different delivery models of pre-operative education would also be of great benefit. Examples of these educational programmes may include: small-group teaching, large-group lectures, individualised one-to-one teaching, online teaching, or mobile device apps. Thorough health economic research is also required to understand the scale of financial savings associated with the routine implementation of pre-operative educational programmes.

## Conclusions

This review suggests that pre-operative patient education is associated with a modest decrease in length of hospital stay following elective orthopaedic surgery in adults, compared with no formal pre-operative education. There may also be a positive association between pre-operative education and anxiety and patient satisfaction. Further research is needed to fully understand the other benefits of pre-operative patient education, to identify which methods of education are most beneficial, and to economically evaluate the practice of pre-operative patient education.

## Appendices

Appendix A

Search Strategies

**Table 5:** Search strategies.

Literature Search Database	Search Terms
OVID Medline search	1 orthop*.ti,ab.
	2 Arthro*.ti,ab.
Ovid MEDLINE(R) and Epub Ahead of Print, In-Process, In-Data-Review & Other Non-Indexed Citations and Daily <1946 to February 27, 2023>	3 (Spin* adj5 surg*).ti,ab.
	4 (Joint* adj5 surg*).ti,ab.
	5 (Spin* adj5 oper*).ti,ab.
	6 (Joint* adj5 oper*).ti,ab.
	7 Decompress*.ti,ab.
	8 exp Orthopedic Procedures/
	9 Orthopedics/
	10 Traumatology/
	11 Educat*.ti,ab.
	12 Teach*.ti,ab.
	13 Taught.ti,ab.
	14 Learn*.ti,ab.
	15 Inform*.ti,ab.
	16 Joint school.ti,ab.
	17 Pre-habilitation.ti,ab.
	18 Prehabilitation.ti,ab.
	19 Class.ti,ab.
	20 exp Health Education/
	21 "Physical Education and Training"/
	22 11 or 12 or 13 or 14 or 15 or 16 or 17 or 18 or 19 or 20 or 21
	23 1 or 2 or 3 or 4 or 5 or 6 or 7 or 8 or 9 or 10
	24 22 and 23
	25 trial.ti,ab.
	26 observational.ti,ab.
	27 randomi*.ti,ab.
	28 25 or 26 or 27
	29 24 and 28
	30 limit 29 to yr="1990 -Current”
OVID Embase search	1 orthop*.ti,ab.
	2 Arthro*.ti,ab.
Embase <1974 to 2023 February 27>	3 (Spin* adj5 surg*).ti,ab.
	4 (Joint* adj5 surg*).ti,ab.
	5 (Spin* adj5 oper*).ti,ab.
	6 (Joint* adj5 oper*).ti,ab.
	7 Decompress*.ti,ab.
	8 exp orthopedic surgery/
	9 exp orthopedics/
	10 exp traumatology/
	11 Educat*.ti,ab.
	12 Teach*.ti,ab.
	13 Taught.ti,ab.
	14 Learn*.ti,ab.
	15 Inform*.ti,ab.
	16 Joint school.ti,ab.
	17 Pre-habilitation.ti,ab.
	18 Prehabilitation.ti,ab.
	19 Class.ti,ab.
	20 exp health education/
	21 exp physical education/
	22 11 or 12 or 13 or 14 or 15 or 16 or 17 or 18 or 19 or 20 or 21
	23 1 or 2 or 3 or 4 or 5 or 6 or 7 or 8 or 9 or 10
	24 22 and 23
	25 trial.ti,ab.
	26 observational.ti,ab.
	27 randomi*.ti,ab.
	28 25 or 26 or 27
	29 24 and 28
	30 limit 29 to yr="1990 -Current”
AMED search	1 orthop*.mp. [mp=abstract, heading words, title]
	2 arthro*.mp. [mp=abstract, heading words, title]
AMED (Allied and Complementary Medicine) <1985 to February 2023>	3 (spin* adj5 surg*).mp. [mp=abstract, heading words, title]
	4 (joint adj5 surg*).mp. [mp=abstract, heading words, title]
	5 (spin* adj5 oper*).mp. [mp=abstract, heading words, title]
	6 (joint adj5 oper*).mp. [mp=abstract, heading words, title]
	7 decompress*.mp. [mp=abstract, heading words, title]
	8 Orthopedics/
	9 educat*.mp. [mp=abstract, heading words, title]
	10 teach*.mp. [mp=abstract, heading words, title]
	11 taught.mp. [mp=abstract, heading words, title]
	12 learn*.mp. [mp=abstract, heading words, title]
	13 inform*.mp. [mp=abstract, heading words, title]
	14 joint school*.mp. [mp=abstract, heading words, title]
	15 pre-habilitation*.mp. [mp=abstract, heading words, title]
	16 prehabilitation*.mp. [mp=abstract, heading words, title]
	17 class.mp. [mp=abstract, heading words, title]
	18 Health education/
	19 Physical education/
	20 1 or 2 or 3 or 4 or 5 or 6 or 7 or 8
	21 9 or 10 or 11 or 12 or 13 or 14 or 15 or 16 or 17 or 18 or 19
	22 20 and 21
	23 trial.mp. [mp=abstract, heading words, title]
	24 observational.mp. [mp=abstract, heading words, title]
	25 randomi*.mp. [mp=abstract, heading words, title]
	26 23 or 24 or 25
	27 22 and 26
CINAHL search	S1 (TI orthop*) OR (AB orthop*)
	S2 (TI orthop*) OR (AB orthop*)
	S3 (TI spin* N5 surg*) OR (AB spin* N5 surg*)
	S4 (TI joint N5 surg*) OR (AB joint N5 surg*)
	S5 (TI spin* N5 oper*) OR (AB spin* N5 oper*)
	S6 (TI joint N5 oper*) OR (AB joint N5 oper*)
	S7 (TI decompress*) OR (AB decompress*)
	S8 SU orthopaedic surgery
	S9 SU orthopaedic
	S10 SU traumatology
	S11 (TI educat*) OR (AB educat*)
	S12 (TI teach*) OR (AB teach*)
	S13 (TI taught) OR (AB taught)
	S14 (TI learn*) OR (AB learn*)
	S15 (TI inform*) OR (AB inform*)
	S16 (TI joint school*) OR (AB joint school*)
	S17 (TI pre-habilitation*) OR (AB pre-habilitation*
	S18 (TI prehabilitation*) OR (AB prehabilitation*)
	S19 (TI class) OR (AB class)
	S20 SU health education
	S21 SU physical education
	S22 S11 OR S12 OR S13 OR S14 OR S15 OR S16 OR S17 OR S18 OR S19 OR S20 OR S21
	S23 S1 OR S2 OR S3 OR S4 OR S5 OR S6 OR S7 OR S8 OR S9 OR S10
	S24 (TI trial) OR (AB trial)
	S25 (TI observational) OR (AB observational)
	S26 (TI randomi*) OR (AB randomi*)
	S27 S24 OR S25 OR S26
	S28 S22 AND S23
	S29 S27 AND S28
PsychInfo search	((tiab(orthop*) OR tiab(arthro*) OR tiab(spin* N/5 surg*) OR tiab(joint N/5 surg*) OR tiab(spin* N/5 oper*) OR tiab(joint N/5 oper*) OR tiab(decompress*) OR subject(orthopaedic surgery) OR subject(orthopaedic) OR subject(traumatology)) AND (tiab(learn*) OR tiab(educat*) OR tiab(teach*) OR tiab(taught) OR tiab(inform*) OR tiab(joint school) OR tiab(pre-habilitation) OR tiab(prehabilitation) OR tiab(class) OR subject(health education) OR subject(physical education))) AND (tiab(trial) OR tiab(observational) OR tiab(randomi*))
WHO clinical trials registry (recruitment status “all”)	(orthop* OR arthro* OR decompress*) AND (educat* OR taught OR learn* OR inform* OR joint school OR pre-habilitation OR prehabilitation OR class)
Clinicaltrials.gov (“completed” and “active, not recruiting” studies only)	(orthopedics OR orthopaedics OR arthroplasty OR arthroscopy OR arthrodesis OR decompression) AND (education OR educate OR taught OR learn OR inform OR information OR joint school OR pre-habilitation OR prehabilitation OR class)
OpenGrey.eu search	((orthop* OR arthro* OR (spin* NEAR/5 surg*) OR (joint* NEAR/5 surg*) OR (spin* NEAR/5 oper*) OR (joint* NEAR/5 oper*) OR decompress*) AND (educat* OR teach* OR taught OR learn* OR inform* OR joint school OR pre-habilitation OR prehabilitation OR class)) AND (trial OR observational OR randomi*)

Appendix B 

Eligibility Criteria

**Table 6:** Eligibility criteria.

1) Is this study written up in English? If no, exclude study.
2) Does this study look at patients aged 18 and over undergoing any elective orthopaedic surgical procedure, for any reason? (to avoid doubt, hand surgery and spinal surgery will be classified as orthopaedic procedures, even if performed by plastics or neurosurgeons) If no, exclude study.
3) Does it compare pre-operative education (see definitions below) with no formal pre-operative education (see definitions below)? (The educational programmes may be delivered and/or arranged by any member of the healthcare team) If no, exclude the study.
4) Is it an experimental or epidemiological/observational comparative study? If no, exclude the study.
5) Does the study measure at least one of the following post-operative outcomes: Length of stay, patient surgical satisfaction, pain scores, functional abilities, adverse events, quality of life, psychological outcomes (anxiety/depression)? If no, exclude the study.
6) Does the study have a retraction statement? If yes, exclude the study, but document the citation for potential inclusion in the discussion.
Definitions: Pre-operative education is defined as any intervention in which a predominant feature is an enhanced level of patient education regarding the anaesthetic, surgery, and recovery period. No formal pre-operative education is defined as a maximum of basic pre-op education only given by the surgeon or a pre-assessment healthcare professional. The study must look solely at pre-operative education; therefore, if the study also uses post-op education as a comparator, exclude it. If the educational intervention focuses on opioid use, exclude it. Exclude studies that focus on pain neuroscience, cognitive, or self-efficacy education.
